# Review of Research Progress on Mo–Si–B Alloys

**DOI:** 10.3390/ma16155495

**Published:** 2023-08-07

**Authors:** Kong Yakang, Cheng Wang, Xiancong Chen, Yi Qu, Jiabo Yu, Haijuan Ju, Xiao Yilei

**Affiliations:** Fundamental Department, Air Force Engineering University, Xi’an 710051, China; kongyakang@126.com (K.Y.); cxcaimath@163.com (X.C.); strsky778@163.com (Y.Q.); b2283216046@163.com (J.Y.); jhjcumtgx@163.com (H.J.); 15979517067@163.com (X.Y.)

**Keywords:** Mo–Si–B alloys, doping and modification, oxidation resistance, mechanical property

## Abstract

Mo–Si–B alloys are a crucial focus for the development of the next generation of ultra-high-temperature structural materials. They have garnered significant attention over the past few decades due to their high melting point and superior strength and oxidation resistance compared to other refractory metal alloys. However, their low fracture toughness at room temperature and poor oxidation resistance at medium temperature are significant barriers limiting the processing and application of Mo–Si–B alloys. Therefore, this review was carried out to compare the effectiveness of doped metallic elements and second-phase particles in solving these problems in detail, in order to provide clear approaches to future research work on Mo–Si–B alloys. It was found that metal doping can enhance the properties of the alloys in several ways. However, their impact on oxidation resistance and fracture toughness at room temperature is limited. Apart from B-rich particles, which significantly improve the high-temperature oxidation resistance of the alloy, the doping of second-phase particles primarily enhances the mechanical properties of the alloys. Additionally, the application of additive manufacturing to Mo–Si–B alloys was discussed, with the observation of high crack density in the alloys prepared using this method. As a result, we suggest a future research direction and the preparation process of oscillatory sintering, which is expected to reduce the porosity of Mo–Si–B alloys, thereby addressing the noted issues.

## 1. Introduction

With the advancement of hypersonic vehicles and advanced aero-propulsion systems, there is a growing need to improve component temperature resistance. In the case of aero-propulsion, fossil fuel combustion in the combustion chamber can generate temperatures over 1700 °C, which exceeds the current operational limits of nickel-based superalloy blades. For this reason, advanced air-cooling system and coatings are used to prevent turbine blades’ degradation and failure. The airflow used to cool turbine blades has now reached 15–20%, as increasing it further could severely damage the efficiency and performance of the engine [1,2,3]. Nickel-based superalloys are currently the most advanced materials for blades. They have been developed over decades and are designed to withstand high operating temperatures of up to 1150 °C, which is near their melting point. To enhance the energy efficiency of gas turbine systems, developing a new generation of ultra-high temperature materials is critical to meet the increasing temperatures of pre-turbine gas. The Mo-Si alloys boast a melting point exceeding 2000 °C and exhibit superior strength and oxidation resistance compared to other refractory metal alloys, making them a highly promising candidate for next-generation ultra-high-temperature structural materials [4].

Mo is a refractory metal with an impressive melting point of up to 2870 K. However, its poor oxidation resistance makes it challenging to use as an independent high-temperature structural material. However, adding Si to Mo can produce a protective silicate glass oxide film at high temperatures, improving its oxidation resistance [5]. If B is added to the alloy, the borosilicate glass scale will form in the initial oxidation stage, providing protection and effectively preventing oxidative degradation under medium temperature conditions [6]. According to a report by Shifler [7], the use of Mo–Si–B alloy in jet engines could lead to fuel savings ranging from 20% to 40%. Kauss performed stress-strain analyses on the turbine blades fabricated from Mo-17.5Si-8B and Mo-9Si-8B alloys, subjecting them to transient thermal-mechanical loads during startup and shutdown of the gas turbine via thermodynamic and structural-mechanical calculations [8]. The findings indicate that the two alloys produced similar results, with the thermal strain being lower by less than 50% compared to CMSX-4, a highly advanced nickel-based superalloy. Additionally, the displacement vectors generated by CMSX-4 turbine blades during startup were over twice those of the Mo-based alloys, suggesting that Mo–Si–B alloys hold broad prospects for use in turbine blade applications. The main focus of current research on Mo–Si–B alloys is on two three-phase regions: Mo_ss_ + Mo_3_Si + MoSiB_2_ (commonly referred to as the Berczik triangle) and Mo_5_Si_3_ + Mo_5_SiB_2_ + Mo_3_Si. Whereas the latter region exhibits compound intermetallic phases with impressive creep resistance and oxidation resistance, the limited fracture toughness of these materials at room temperature (2–4 MPa∙m^1/2^) limits their use as structural materials [9]. Nevertheless, the region of the Berczik triangle, which comprises the ductile Mo_ss_ (Mo solid solution) phase as well as two compound intermetallic phases, exhibits better comprehensive performance, motivating current research efforts in the area of Mo–Si–B alloys [10,11]. 

In his 2003 review, Dimiduk noted that the high density, low oxidation resistance at medium temperature and poor toughness of Mo–Si–B alloys pose significant challenges for their use in replacing nickel-based superalloys in turbine engines [12]. Extensive research over the last thirty years has focused on studying the effects of doping, phase composition, and phase ratios, as well as preparation techniques, on the properties of Mo–Si–B alloys. However, numerous studies on modifying Mo–Si–B alloys tend to focus only on a certain point with varying emphases, and different modification techniques produce differing effects on enhancing alloys properties, so it is necessary to summarize the regularity of many modification methods. In order to optimize the balance between creep, toughness, and oxidation resistance in Mo–Si–B alloys, Brindley conducted a design study on the phase fraction, and pointed out that when the faction of Mo_ss_ is 55% to 63%, the mechanical properties can be balanced [13]. However, it must be noted that a greater amount of Mo_ss_ is detrimental to the oxidation resistance. Zhao and Pan have conducted numerous studies on the characteristics of Mo-Ti-Si-B alloys and the oxidation behavior of Mo–Si–B alloys, respectively, and reviewed the relevant studies [14,15]. Although these studies have advanced our understanding, there is still a lack of information on the effects of metal elements, second-phase particle doping, and microstructure scale design modification on the properties of Mo–Si–B alloys. Based on this reality, the authors here draw on recent research findings to provide an overview of the effects of metal elements and second-phase particles’ modification, and the proportion of each phase in Mo–Si–B alloys, on their properties. Additionally, the paper summarizes the current state of additive manufacturing technology for the production of Mo–Si–B alloys. These comprehensive understandings can be used as references for future research in this area. Finally, the research presents potential methods and preparation techniques for enhancing the properties of these alloys in the future. 

## 2. Mo–Si–B Alloy

The Mo–Si–B ternary alloys exhibit brittleness around 1000 °C and typically remain brittle at room temperature. Mo_ss_, acting as a ductile phase, primarily governs the deformation response of the alloy and can augment its toughness by enabling the passivation and trapping of cracks. The continuous dispersion of the Mo_ss_ coarse grains can increase the fracture toughness and fatigue life of the alloys, with a range of fracture toughness from about 5 MPa∙m^1/2^ to 20 MPa∙m^1/2^ as a function of Mo_ss_ volume fraction [16,17]. Krüger et al. used the mean field homogenization method to study the effect of the matrix type of the multiphase Mo–Si–B alloy on stress–strain behavior. In the process of high-temperature deformation, there is a high stress in the silicide phases, and the deformation performance of the alloys is mainly determined by the Mo_ss_ phase [18]. However, due to the ease with which Mo is oxidized to MoO_3_ and volatilized, the Mo_ss_ phase exhibits poor oxidation resistance. Multiple studies have indicated that the oxidation resistance of Mo–Si–B alloy is also poor in the three-phase region, with high Mo content at temperatures of 1300 °C or above [19,20,21,22]. Intermetallic compounds have good creep resistance as strengthening phases. Among them, phase A15 (Mo_3_Si) possesses the high atomic weight of Mo, poor oxidation resistance, and a lower high-temperature strength than phase T2 (Mo_5_SiB_2_) and phase T1 (Mo_5_Si_3_), which makes it an unsatisfactory phase in alloys. Ochiai investigated the oxidation behavior of monolithic Mo_3_Si specimens at 900 °C and observed mass loss of up to 120 mg/cm^2^ within the first 1–2 h of the initial oxidation stage [23]. This loss was predominantly attributed to the generation and subsequent vaporization of MoO_3_, resulting in the depletion of the material. However, as a supplier of Si, Mo_3_Si also plays a role in improving the oxidation resistance at 1000–1300 °C [21,24]. In contrast, the T2 phase boasts an incredibly high melting point (over 2200 °C), a relatively low density (8.864 g/cm^3^), and exceptional high-temperature strength and stress deformation resistance. Specifically, its creep rate at 1300 °C is three orders of magnitude lower than that of the most rigid [001]-orientation MoSi_2_ single crystals, making it ideal for alloys [25,26,27]. The mechanical characteristics of alloys are influenced not only by the composition and distribution of each phase, but also by grain size. Lemberg et al. discovered that the fracture toughness of a fine-grained microstructure is lower than that of a coarse-grained one because the plastic deformation mechanics of the alloy change from intracrystalline slip to intergranular slip due to a reduction in grain size [3]. Conversely, grain size decreases and grain boundary increases, which effectively prevents the dislocation movement and improves the alloy’s strength. To account for the alloy’s mechanical properties and oxidation resistance, typically no more than 50% Mo_ss_ is included. The Mo-12Si-8.5B alloy strikes a suitable balance with a volume fraction of around 40% Mo_ss_ and 60% intermetallic phases, offering both mechanical strength and oxidation resistance [28,29]. However, the alloy still suffers from poor fracture toughness, which hinders wide-scale application and manufacture. 

The reason for Mo–Si–B alloys’ excellent oxidation resistance in the temperature range of 900 °C to 1300 °C is due to the formation of the borosilicate scale, which effectively isolates oxygen and reduces the reaction rate between oxygen and the inner metal. However, at the moderate temperature of 800 °C, the oxidation rate accelerates rapidly, leading to the formation of a volatile oxide layer. In severe cases, catastrophic “pesting” oxidation can occur, causing the alloy to collapse quickly [25,26]. The oxidation behavior of Mo–Si–B varies with the decrease in oxygen pressure. During the initial oxidation stage with high oxygen partial pressure, the reaction proceeds according to Equations (1)–(3). As the borosilicate layer (B_2_O_3_-SiO_2_) forms and thickens, the oxygen partial pressure decreases, leading to a reaction following Equations (4)–(6). As the slow oxidation proceeds and the oxygen partial pressure is further reduced, the oxidation reaction continues according to Equations (7)–(8) [15,20,30,31].

2Mo + 3O_2_ = 2MoO_3_ (1)

2Mo_3_Si + 11O_2_ = 6MoO_3_ + 2SiO_2_ (2)

Mo_5_SiB_2_ + 10O_2_ = 5MoO_3_ + SiO_2_ + B_2_O_3_ (3)

Mo + O_2_ = MoO_2_ (4)

Mo_3_Si + 4O_2_ = 3MoO_2_ + SiO_2_ (5)

2Mo_5_SiB_2_ + 15O_2_ = 10MoO_2_ + 2SiO_2_ + 2B_2_O_3_ (6)

Mo_3_Si + O_2_ = 3Mo + SiO_2_ (7)

2Mo_5_SiB_2_ + 5O_2_ = 10Mo + 2SiO_2_ + 2B_2_O_3_ (8)

Parthasarathy et al. and Rioult et al. described the process as a two-stage process [19,32]. In the transient stage, MoO_3_, which is the product of reaction (1), evaporates at temperature above 475 °C; as reactions (2) and (3) occur, the borosilicate forms and gradually covers the alloys surface, preventing the volatilization of MoO_3_ and cutting off oxygen. When the borosilicate scale covers the entire alloys surface, the process enters the steady-state oxidation stage. The mass loss of the alloys during oxidation mainly results from the volatilization of MoO_3_. Typically, this accounts for more than half of the total mass loss in extended oxidation tests. Meanwhile, the mass increase occurs mainly through the formation and adhesion of SiO_2_ and B_2_O_3_. Pan noted in his review that the shape of the TG curve is influenced by the coupling of the two kinds of mass change [15]. Therefore, the mass variation during oxidation may not accurately reflect the oxidation behavior of the alloys. Therefore, analyzing both the microstructure evolution and the oxide layer is crucial to understanding the oxidation process of Mo–Si–B alloys. As previously indicated, the Mo_ss_, A15, and T2 phases in the three-phase Mo–Si–B alloys serve to mitigate each other’s deficiencies, but also pose challenges to enhancing the alloys ductility and environmental adaptability due to their mutual constraints. The volume fraction, distribution, and grain size of each phase significantly affect the overall performance of the alloys. A finer and more uniform distribution of intermetallic compounds leads to the faster formation of borosilicate scale, and smaller grain sizes result in shorter distances for transverse growth during oxidation [19]. Moreover, optimal borosilicate viscosity can provide rapid and efficient protection for the substrate in the oxidizing environment. Thus, there are two primary approaches to enhancing the oxidation resistance of Mo–Si–B alloy. Firstly, one may regulate the microstructure by minimizing the proportion of Mo_ss_ and refining the grain [24]. Secondly, the other option is to modify the borosilicate scale. Research has shown that achieving a uniform distribution of each phase is crucial in balancing strength, increasing creep resistance, and enhancing ductility [33,34]. As a result, the mechanical properties of alloys can be improved through microstructure control and structural design, such as ultra-fine grain design and double-scale grain design [35,36]. As such, many researchers have conducted extensive studies on the impact of active metal elements such as Zr [37,38], Y [30], Ti [14,39] and Al [31,40], along with second-phase particles such as La_2_O_3_ [28,41] and ZrB_2_ [22], on Mo–Si–B alloys to enhance their overall properties.

## 3. Metallic Elements Modified Mo–Si–B Alloys

Previous research has shown that the grain boundaries serve as the primary pathway for oxygen diffusion. The introduction of active elements such as Zr [37], Nb [42,43], Al [31,44], and Ti [39] into the alloy has been shown to effectively absorb oxygen at the grain boundaries, forming oxides and reducing the oxidation rate of the alloys. In addition, these oxides, which are firmly adhered to the grain boundaries, can enhance their strength and prevent the oxidation film from detaching. This will improve the alloys’ toughness and oxidation resistance. The high temperature deformation behavior of the alloy is influenced by microstructure size, while atomic diffusion and dislocation slip are affected by both grain size and grain boundary strength. Although Si can enhance the high-temperature strength of Mo–Si–B alloys through solution strengthening, the concentration of Si and O elements at the grain boundary can weaken grain boundary cohesion, leading to intergranular fracturing over a wide range that can seriously affect the toughness of the alloy [45,46]. The addition of trace Zr not only maintains the phase composition but also reduces the segregation of Si and O to the grain boundary, thus enhancing the toughness of the alloy. Furthermore, Hochmuth’s research suggested that Zr inhibits SiO_2_ formation at grain boundaries, leading to improved grain boundary strength and a significant reduction in the creep rate of Mo-9Si-B alloys [47]. The introduction of trace amounts of Zr not only leaves the phase composition unchanged, but also mitigates the segregation of Si and O towards the grain boundary, thus enhancing the toughness of the alloy [48].

### 3.1. Al Element Modified Mo–Si–B Alloys

**Figure 1:**
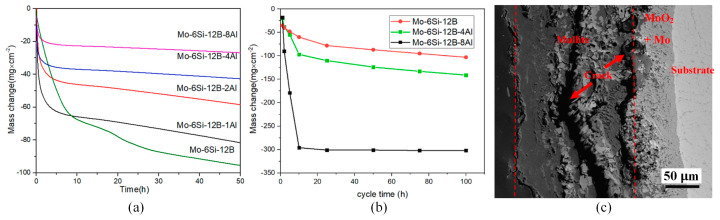
(**a**) Mass loss of Mo-6Si-12B-(0, 1, 2, 4, 8) Al samples during isothermal oxidation at 1300 °C. (**b**) Mass loss of Mo-6Si-12B-(0, 4, 8) Al samples during cyclic oxidation at 1300 °C. (**c**) Oxidation layer morphology of Mo-6Si-12B-8Al sample after cyclic oxidation at 1300 °C for 10 h [55].

It is widely recognized that the inclusion of Al in alloys can generate a compact Al_2_O_3_ oxygen diffusion barrier and strengthen the oxidation resistance of superalloys [31,49,50,51]. However, Paswan’s research yielded contrasting results. He discovered that the oxidation resistance of Mo-based superalloys diminished with the incorporation of Al during the isothermal oxidation tests conducted at 400~800 °C, and mullite tended to become less dense and more permeable in the cyclic oxidation test, which allowed for oxygen diffusion into the alloy [31,52,53]. Rosales’s research revealed that Mo_3_Si alloy samples exhibited severe “pesting” oxidation in the air at 1000 °C, and the addition of Al resulted in a typical oxidation behavior characterized by initial rapid weight loss followed by a steady state of little weight change [54]. This can be attributed to the protective effect of Al_2_O_3_ and SiO_2_. In a recent study, Liu et al. investigated the oxidation behavior of Mo-6Si-12B alloy with varying levels of Al doping (1, 2, 4, 8 at.%) between 800 and 1300 °C [55]. The results of these studies shed light on how Al modifies the properties of Mo–Si–B alloys. The TGA results for isothermal oxidation, as shown in Figure 1a, demonstrate that the addition of Al results in an improvement in the oxidation resistance of the alloy, with a positive correlation between resistance and Al content. The cyclic oxidation test, on the other hand, has revealed that the addition of Al reduces the oxidation resistance of the alloy, with microscopic characterization revealing that the oxidation layer consisted of a mixture of borosilicate and mullite when the Al content was 4 at.%. The percentage of mullite in the oxide layer also increased with increasing Al content, and when the Al content reached 8 at.%, the structure of the outer layer consisted entirely of mullite. During the cyclic oxidation test, repeated cooling and heating cycles may cause cracks to appear in the oxide layer as a result of thermal stress. Unlike the glassy phase, however, mullites do not show self-healing properties. These cracks may act as channels for the oxygen molecules to enter (as shown in Figure 1c), resulting in the continued oxidation of the substrate. Therefore, while the glass and mullite layer provided double-layer protection for the alloy in an isothermal oxidation environment, mullites’ tensile strength may not be able to withstand the impact stress and cracks during cyclic oxidizing conditions. This leaves the alloy exposed to air again. After analyzing the application environment of structural materials, it is concluded that the addition of Al to Mo–Si–B alloys may not be ideal for components such as turbine blades that experience prolonged harsh thermal shock. However, it is an effective method to enhance oxidation resistance for components working in a stable isothermal environment over an extended period.

### 3.2. Nb Element Modified Mo–Si–B Alloys

Since the T2 phase is the only ternary compound present in both three-phase regions, it shows excellent oxidation resistance at medium temperature and creep resistance at high temperature. Liu’s research showed that the oxidation resistance of the Mo–Si–B alloys increased significantly with increasing T2 phase content (6.5–76.0%) [56]. These observations further emphasize the crucial role played by the T2 phase in increasing the alloy’s resistance to oxidation [19]. The oxidation byproduct of Nb has been shown in previous studies to be porous Nb_5_O_2_. In addition, the rate of oxygen diffusion in Nb_5_O_2_ (8.7 × 10^−11^ cm^2/s^) at a temperature of 1000 °C is nearly 1000 times greater than that of silica glass (1.8 × 10^−14^ cm^2/s^), which leads to a decreased protective ability of the borosilicate layer [3,43,57]. In addition to increasing toughness, however, the inclusion of Nb also stabilized the T2 phase [58,59], and various types of research indicate that refractory metallic elements such as Nb lead to the destabilization of the A15 phase, resulting in the BCC + T2 + T1 alloys of the three-phase region [60,61]. It has excellent mechanical properties while retaining oxidation resistance. The impact of the inclusion of Nb on the characteristics of the Mo–Si–B alloys, therefore, continues to be of importance, and the main concern is to identify the minimum amount of Nb added that can effectively suppress the A15 phase. Based on research, Yang suggested that the optimal range for the critical value of Mo-12Si-10B alloy lies between 24 at.% and 26 at.% [62]. The Mo_3_Si phase was absent in the alloy containing 26 at.% of Nb. Subsequently, the density, mechanical properties, and oxidation resistance of Mo-12Si-10B and Mo-26Nb-12Si-10B alloys were compared [42]. By utilizing an identical preparation technique, the porosity for the two samples was 2.5% and 0.2%, correspondingly. The increased density also shifted the alloy from intergranular fracture to transgranular fracture, resulting in an increase in the room temperature fracture toughness from 6.77 ± 0.20 MPa·m^1/2^ to 8.84 ± 0.17 MPa·m^1/2^. In Mo–Si–B alloys, Mo_ss_ is the most ductile and susceptible to deformation at elevated temperatures, and its deformation characteristics play a crucial role in determining the material’s strength at high temperatures, while the incorporation of Nb atoms into the alloy can substantially enhance the compressive strength through solid solution strengthening. After being subjected to oxidation at a temperature of 1300 °C for a duration of 5 h, the Mo-12Si-10B alloy demonstrated the formation of a continuously dense SiO_2_ oxide layer that exhibited a thickness of approximately 75 μm. In contrast, the Mo-26Nb-12Si-10B alloy produced an oxide layer featuring a loose Nb_2_O_5_ structure, which was approximately 1.6 mm thick.

### 3.3. Ti Element Modified Mo–Si–B Alloys

When it comes to microstructure optimization, it has been increasingly recognized that Ti addition can decrease alloy density and enhance oxidation resistance while maintaining fracture toughness. More importantly, the oxidation phenomenon of “pesting” at medium temperature is inhibited in some Mo-Ti-Si-B composites. The main reason is that the macroscopic alloying of Ti can increase Si concentration without losing the ductile phase, which is precisely what the ternary Mo–Si–B alloys cannot achieve [63,64,65,66,67,68,69]. Zhao et al. produced Mo-Ti-Si-B alloys with three different microstructure components by arc melting [70]. Their study showed that the volume fraction of Mo_3_Si in the sample decreased with increasing Ti content and even disappeared completely in the 30Mo-40Ti-20Si-10B alloy with a phase composition of Mo_ss_ + Mo_5_SiB_2_ + Ti_5_Si_3_. Based on previous research findings, monolithic Ti_5_Si_3_ demonstrates exceptional resistance to oxidation in the air up to 1250 °C [71,72,73,74]. As the unsatisfactory Mo_3_Si phase gradually disappeared and the content of the Ti_5_Si_3_ phase increased, the alloy oxidation resistance at 800 °C was improved. Specifically, the sample 30Mo-40Ti-20Si-10B exhibited a total mass loss of less than 2 mg/cm^2^ over 25 h. However, summarizing previous studies, the oxidation resistance of Ti-doped Mo–Si–B alloys proved to be temperature- and time-sensitive. At lower oxidation temperatures, resistance depends on the high viscosity of the borosilicate layer, which effectively prevents the evaporation of MoO_3_. However, as time progresses and temperatures rise, the outward diffusion of Ti accelerates. At the same time, a large amount of TiO_2_ is generated in the oxide layer, which destroys the integrity of the borosilicate layer, resulting in the re-establishment of the MoO_3_ volatilization channels and oxygen diffusion in the high-temperature environment [39,75].

**Figure 2:**
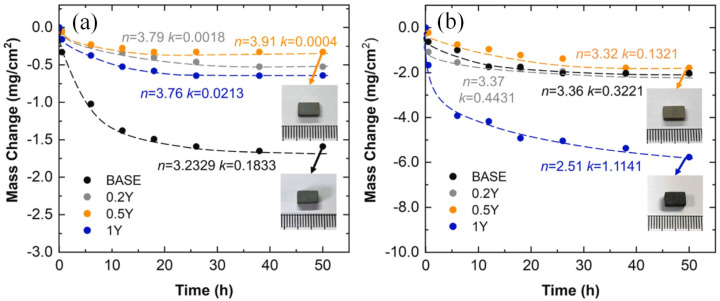
(**a**,**b**) The specific mass change of Mo-40Ti-20Si-10B samples doped with different contents of Y with oxidation time at 800 °C and 1100 °C [76].

Majumdar et al. reported that incorporating Y effectively enhanced the oxidation resistance of Mo-9Si-8B alloy across a wide temperature range [30,76]. In light of this, Gui investigated the microstructure and oxidation characteristics of Mo-Ti-Si-B alloys with varying Y levels (0.2–1.0 at.%) [77]. With the increase in Y content, the grain structure became coarser and the volume fraction of Mo_ss_ increased, while the volume fraction of Ti_5_Si_3_ decreased. Depending on the formation of the outer layer of thermally stable yttrium molybdate (Y_2_MoO_12_), the oxidation resistance of the sample increased significantly at 800 °C, as shown in Figure 2a. However, during oxidation at 1100 °C, Y_2_MoO_12_ will decompose into Y_2_TiO_7_, negatively impacting the oxidation resistance of Mo-Ti-Si-B alloys. Therefore, doping with Y cannot solve the problem of insufficient oxidation resistance at high temperatures and may even worsen the mass loss of the sample at high addition amounts, as illustrated in Figure 2b. Gaitzsch et al. prepared Mo-25Ti-9Si-8B alloy by powder metallurgy [78]. The density of the Mo-25Ti-9Si-8B alloy was only 7.9 g/cm^3^, which reached the density value of general superalloys. During high-temperature processing, a portion of silicon dissolves into the solid solution phase of Mo. However, upon cooling, due to reduced solubility, some Si diffuses to the grain boundary, resulting in silicide precipitates. This process commonly initiates cracks, leading to the embrittlement of Mo–Si–B alloys [79,80]. After heat treatment, the Mo-25Ti-9Si-8B alloy sample experienced the precipitation of Ti_5_Si_3_ particles that were finer than silicates. These particles strengthened the alloy at its grain boundaries. Furthermore, trapping Si in Ti_5_Si_3_ helped to decrease the extent of Si concentration at the alloy grain boundaries, thus enhancing the alloy’s ductility.

In their studies, Zhang et al. explored the impact of various sintering temperatures and Y doping levels (0.1–0.5 at.%) on the microstructure and high-temperature oxidation behavior of a Mo-13Si-25B alloy [81]. At a sintering temperature of 1750 °C, the T2 phase conversion rate was at its highest, with a volume fraction of 98.60%. As the temperature increased, the melting of Si led to the formation of additional MoB and MoSi_3_, causing changes in sample composition. During the initial oxidation stage, Y addition promotes the oxidation of Si, leading to the formation of SiO_2_ and B_2_O_3_ that cover the surface of the substrate. This layer effectively prevented the further oxidation of the material. Furthermore, the solid–liquid reaction of Si and Y at high temperatures produced the monoclinic phase Y_2_Si_2_O_7_, which can be used as a high-temperature oxidation-resistant coating. This coating helps to repair internal cracks and micro defects. The grain size of the alloy is a key factor affecting the oxidation resistance of Mo–Si–B alloys. Finer grain sizes can effectively reduce the distance of the transverse growth of borosilicate, thus shortening the transition time from the transient oxidation stage to the steady oxidation stage, and ultimately minimizing mass loss [82,83]. The Mo-13Si-25B alloy experienced a refinement in its average grain size from 1.44 μm to approximately 400 nm with the addition of 0.2 at.% Y, leading to its superior oxidation resistance. However, excessive doping resulted in a decrease in the T2 phase content, resulting in a decrease in the oxidation resistance of the sample.

To address the issue of reduced ductility and toughness when Si is dissolved in Mo_ss_, Sturm et al. developed a novel alloy system based on Mo_ss_ + Mo_2_B + T2 phases [46]. The low solubility of Si in the Mo_ss_ phase gave this alloy greater fracture toughness while maintaining the same oxidation resistance as the Mo_ss_ + T2 + Mo_3_Si three-phase region alloys. Su et al. then designed a Mo-6Si-12B-4Al-20Ti alloy sample with a density of only 7.9 g/cm^3^ [84]. The microstructure of the sample was composed of Mo_ss_ dendrite solid solution and T2 phase between dendrites. After annealing at 1800 °C, the Si content in the Mo solid solution was reduced to 1.81 at.%, which reduced the hardening effect of Si on the alloy and enhanced the ductility of the alloy. In isothermal environments of 1100 °C and 1200 °C, the sample exhibits excellent oxidation resistance. However, at 1300 °C, the sample displays a moderate amount of mass loss after undergoing oxidation for 50 h, with no cessation of oxidation observed. The results of a cyclic oxidation test conducted at 1300 °C using a 1 h furnace and 15 min exterior method indicate that the Mo-6Si-12B-4Al-20Ti sample exhibited a smaller mass loss than the Mo-6Si-12B sample within 50 h. However, the former continued to undergo oxidation beyond 50 h, while the latter showed almost no mass loss. For the Mo-6Si-12B-4Al-20Ti alloy, Ti and Al are beneficial elements that facilitate the formation of thermodynamically stable oxides. The oxide layer is primarily composed of TiO_2_, with a small amount of Al_2_O_3_, which is more protective than the borosilicate scale of the Mo-6Si-12B sample. However, as the temperature increases, Ti atoms diffuse outward at a higher rate. The author attributed the decline in sample oxidation resistance at 1300 °C to the high vapor pressure of TiO_2_ (1.3 × 10^−2^ Pa), which can hurt the long-term oxidation resistance of the oxide layer. In summary, Ti doping can offer several advantages, such as reducing the density of Mo–Si–B alloys to an acceptable level, preventing the formation of unsatisfactory phase A15, and inhibiting Si segregation to the grain boundary by forming Ti_5_Si_3_ in the solid solution phase of Mo, thus enhancing the ductility of the alloy. However, a significant amount of TiO_2_ generated in the oxide layer may compromise the integrity of the protective layer, resulting in insufficient oxidation resistance at temperatures beyond 1300 °C.

## 4. Mo–Si–B Alloys Modified by Second Phase Particle

During the preparation process, molybdenum alloys inevitably contain impurities such as oxygen or nitrogen. As these impurities gather at grain boundaries, they form local oxides and bubbles that can decrease the toughness and strength of the alloy by weakening its grain boundaries. The doping of the second-phase particles can strengthen the alloy by preventing dislocation or grain boundary sliding and refining the grain, and can also improve the oxidation resistance of the alloy by limiting the penetration and diffusion of oxygen atoms. Grain refinement not only enhances alloy strength, but also increases the number of grain boundaries, minimizes stress concentration within the crystal, and retards the alloy’s plastic deformation process.

### 4.1. Mo–Si–B Alloys Modified by ZrB_2_

**Figure 3:**
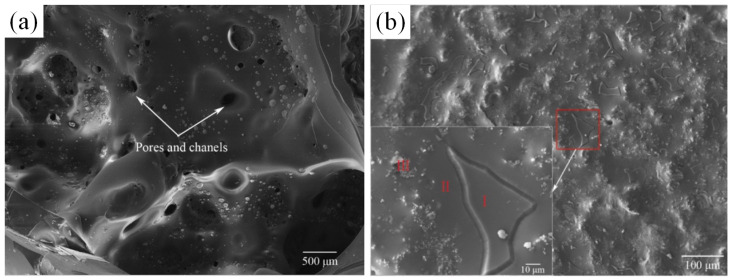
SEM image of the surface of borosilicate layer formed after 27 h oxidation in air at 1400 °C: (**a**) Mo-12Si-8.5B alloy; (**b**) Mo-12Si-8.5B alloy with 2.5 wt. % ZrB_2_ added [86].

Wang conducted an in-depth study of the effect of ZrB_2_-doped particles on the Mo-12Si-8.5B alloy, firstly exploring the effects of different doping levels on the mechanical properties of the alloy [22]. The results show that increasing or decreasing the amount of doping has different effects on the strength and toughness of the alloy. During the sintering process, ZrB_2_ can adsorb oxygen and form ZrO_2_ particles, which limits SiO_2_ formation at grain boundaries and enhances the grain boundary bonding strength of the alloy. Additionally, the pinning effect of ZrO_2_ particles on dislocations promoted the accumulation of said dislocations in Mo_ss_ grains, leading to mutual interference that increases the toughness of the alloy. During sintering, dispersed ZrO_2_ particles blocked the further growth of grains, resulting in a stable ultrafine microstructure ranging in size from 0.47 μm to 0.81 μm. Therefore, the increase in alloy strength can be attributed to the formation of an ultrafine grain structure. As ZrB_2_ contents increased, the grain size was gradually refined, resulting in a continuous increase in the alloy strength. On the contrary, increasing the amount of doping will lead to more particles reaching the grain boundary, resulting in stress concentration during large-strain plastic deformation, so the doping of ZrB_2_ achieves an optimal content for enhancing toughness. Specifically, the fracture toughness reached 11.5 MPa∙m^1/2^ with the doping of 1.0 wt. % ZrB_2_, and the further increase leads to a decrease in fracture toughness. Therefore, the alloy doped with 1.0 wt. % ZrB_2_ showed the best combination of toughness and strength. Based on these findings, Wang et al. conducted additional investigations into the oxidation characteristics of a Mo-12Si-8.5B alloy that was enhanced with 1.0 wt. % ZrB_2_ [85], with a focus on its performance at 1300 °C. The borosilicate within the alloy exhibited a finer microstructure and improved fluidity, resulting in the alloy reaching the steady-state oxidation stage in only 300 s, as opposed to 2.7 h for the alloy lacking ZrB_2_ addition. Moreover, a multitude of ZrO_2_/ZrSiO_4_ particles was generated during oxidation and randomly distributed within the borosilicate layer, thereby enhancing its effectiveness in protecting during the stable oxidation phase. The author subsequently raised the ZrB_2_ content to 2.5 wt. % and assessed the oxidation resistance of the specimens at a temperature of 1400 °C [86]. In addition to reactions (1) to (3), the oxidation process depicted in formula (9) was also observed during the transient oxidation phase of the ZrB_2_-doped alloy. As B_2_O_3_ production increased, the viscosity of the borosilicate scale rich in SiO_2_ decreased, facilitating the rapid coating of the borosilicate scale. In the steady-state oxidation phase at a temperature of 1400 °C, as depicted in Figure 3a, the borosilicate layer with low viscosity failed to efficiently impede the diffusion of oxygen molecules. Consequently, the substrate underwent continuous oxidation, resulting in the creation of additional pores as MoO_3_ evaporated, forming a vicious cycle. However, for samples containing ZrB_2_, the surface silicate borate glass layer was enriched with Zr. As a glass network-forming agent [87], Zr restricted the flowability of SiO_2_ glass at high temperatures and accelerated the passivation of the silicate borate glass layer. As a result, the ZrB_2_-doped alloy exhibited a more compact oxide layer at elevated temperatures, demonstrating excellent oxidation resistance, as illustrated in Figure 3b. However, in Wang’s recent study [88], it was found that doping ZrB_2_ did not effectively enhance the oxidation resistance of Mo-12Si-8.5B alloy at 900 °C, even with a doping amount as high as 2.5 wt. %. The sample doped with ZrB_2_ exhibited stable protection against oxidation at 1300 °C, thanks to the formation of a protective oxide layer on its surface. Inspired by this, Wang subjected the sample to pre-oxidation at 1300 °C followed by oxidation at 900 °C. The borosilicate layer formed on the pre-oxidized sample surface was both dense and continuous, which resulted in a reduced initial mass loss during the oxidation process at 900 °C. However, over time, the accumulation of MoO_3_ could cause the oxidation layer to rupture, ultimately accelerating the oxidation behavior of the sample. As such, while pre-oxidation may improve oxidation resistance, its effectiveness is ultimately limited.

2ZrB_2_ + 5O_2_ = 2ZrO_2_ + B_2_O_3_ (9)

### 4.2. Mo–Si–B Alloys Modified by La_2_O_3_

As far back as 2013, Zhang employed a solid–solid doping technique to fabricate Mo-12Si-8.5B alloy with varying amounts of La_2_O_3_ [89], resulting in a boost in its compressive strength to 2.7 GPa, but the toughening effect was not obvious. The main mechanisms were fine-grain reinforcement and particle dispersion reinforcement. Conventional doping methods tended to introduce rigid particles into the grain boundary, causing stress concentration and localized crack propagation near the boundary. While grain refinement can increase yield strength, its ductility is constrained by crack formation. To tackle this issue, Liu utilized molecular liquid–liquid mixing/doping technology to uniformly disperse La_2_O_3_ throughout the grain [90]. This led to a 400% improvement in the fracture toughness of the alloy since the doped particles within the grain can generate, stabilize, and accumulate dislocations within the material. Then, Li used the above method to incorporate variable mass percentages of La_2_O_3_ into the Mo-12Si-8.5B alloy [28]. As the content increased, the grain size of the alloy was progressively refined, thus increasing its compressive strength to 2.97 GPa. Li conducted a parametric investigation into the amount of La_2_O_3_ doping, and discovered that the reinforcement effect was highly responsive to the added amount [91], with the optimal addition amount being 0.9 wt. % when the alloy strength was at its highest. However, the toughening effect remained insignificant. Simultaneously, he pointed out that excessive addition not only fails to refine the grain size but also causes a decline in the mechanical properties of the alloy. This occurred primarily due to the presence of excessive particles at the grain boundaries of the alloy, which generated an abundance of microcracks during the deformation process. Nevertheless, Zhang’s findings indicate that increasing the La_2_O_3_ concentration, despite yielding only moderate enhancement in the alloy strength, does not cause any deterioration in its mechanical properties [89]. Both researchers used the same preparation technology and parameter values, and the reasons for inconsistent conclusions remain uncertain. However, Li’s findings appear to be more in line with reality [91]. The hard particles at the grain boundary found by Liu in the study will cause intergranular cracks, which also indicate that excessive doping may damage the alloy’s mechanical properties.

Cheng et al. conducted creep tests on Mo samples with and without the addition of La_2_O_3_ particles at 1300 °C/60 MPa [41]. Compared to Mo-La_2_O_3_ alloy, pure Mo exhibited a much higher steady-state creep rate under the same creep stress. Furthermore, the amount of La_2_O_3_ increased from 0.6 wt. % to 1.5 wt. %, and the steady-state creep rate of the sample decreased by almost an order of magnitude. The constitutive model, which relied on the interaction between dislocations and particles, indicates that the primary creep reinforcement mechanism was the low relaxation efficiency of dislocation line energy. After many experiments, the fracture patterns of Mo-La_2_O_3_ alloy at different temperatures/creep rates was summarized by Cheng. When the creep rate is high and the creep temperature is low (less than 1300 °C), necking occurs, resulting in a fracture morphology characterized by significant dislocation-induced plastic deformation, seen as numerous dimples. Decreasing the creep rate leads to the activation of GBS as a secondary deformation mechanism, resulting in a brittle cleavage fracture and mixed fracture mode. When the creep rate is low (less than 10^−7^ s^−1^), transgranular fracture is the primary fracture mode.

### 4.3. Mo–Si–B Alloys Modified by Carbide and Oxide

Carbide and oxide whiskers/fibers are often used to enhance the toughness of metals, intermetallic materials, or ceramics [92,93]. Li et al. incorporated SiC whiskers into alloy powder by liquid-liquid doping and fabricated the alloy by hot pressing [94]. They discovered that the SiC addition enabled the regulation of phase composition and led to a substantial increase in the intermetallic compound content. On this basis, the effects of SiC and TiO_2_ addition on the oxidation resistance of Mo-12Si-8.5B alloys were compared by Li [95]. In the oxidizing environment at 1300 °C, the dynamic curves for the alloys both with and without whiskers were identical. The addition of whiskers effectively reduced the time and overall mass loss required for the transient oxidation stage. Compared to other alloys, the Mo–Si–B-SiC alloy experienced lower mass loss. However, in the oxidizing atmosphere at 1400 °C, the Mo–Si–B-TiO_2_ alloy experienced rapid weight loss in the initial stage followed by entering the steady-state phase. While the total mass loss was significant, there was negligible mass loss during the steady-state phase. To clarify these phenomena, the authors analyzed the oxidation scale. The oxidation scale of the Mo–Si–B-TiO_2_ alloy consisted of SiO_2_, TiO_2_, and B_2_O_3_ at different temperatures. At 1300 °C, the oxidation scale was nonporous, but at 1400 °C, numerous pores appeared on the surface of the oxidation scale. Aligned with the Ti doping effects on the alloy, TiO_2_ as a glass mesh modifier can effectively decrease SiO_2_ viscosity. At lower temperatures, the low-viscosity borosilicate scale rapidly covered the substrate, resulting in the Mo–Si–B-TiO_2_ alloy having the shortest transient oxidation time. However, as the temperature rose, the low-viscosity borosilicate scale failed to obstruct oxygen penetration, and the evaporation of MoO_3_ created holes that led to rapid weight loss during the transient oxidation stage at 1400 °C. For Mo–Si–B-SiC alloy, the reaction of Formulas (10) and (11) provided a large amount of SiO_2_ for oxidation scale formation, so that a stable protective layer can be formed under two oxidation environments at different temperatures. Moreover, the high Si/B ratio of borosilicate on the sample surface promoted the rapid passivation of the borosilicate layer at high temperatures.

2SiC + 3O_2_ = 2SiO_2_ + 2CO (10)

2SiC + 3O_2_ = 2SiO_2_ + 2CO (11)

### 4.4. Mo–Si–B Alloys Modified by MAX Phase

**Figure 4:**
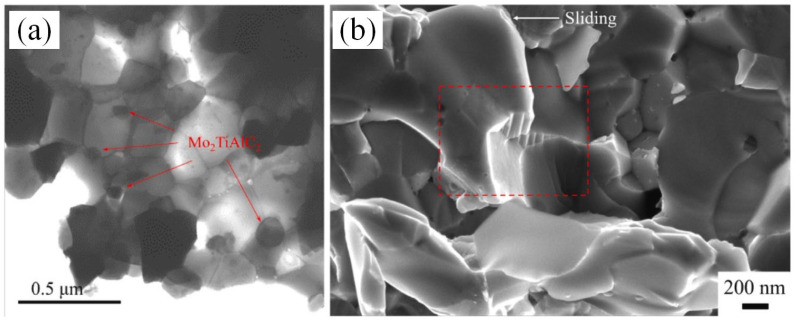
(**a**) TEM image of microstructure of alloy; (**b**) a continuous stepped structure formed by the fracture or sliding of Mo_2_TiAlC_2_ particles. The red rectangle in the picture frames the ladder structure [99].

MAX phase is a layered structure that has the potential to enhance fracture toughness and strength in ceramics and intermetallic compounds [96,97]. In 2015, Anasori et al. successfully synthesized an ordered Mo-based MAX phase, Mo_2_TiAlC_2_, for the first time. Such a layered structure can consume crack propagation energy under stress conditions through twisting and delamination [98]. Based on this, Lin et al. conducted a study on enhancing the mechanical properties of the Mo-12Si-8.5B alloy by incorporating the Mo_2_TiAlC_2_ phase [99]. Of the three phases of the Mo-12Si-8.5B alloy, the Mo_ss_ phase has the lowest hardness of about 200–300 HV [9,100], while the hardness values of Mo_3_Si and Mo_5_SiB_2_ are about 1316 HV and 1836 HV, respectively [101]. Mo_2_TiAlC_2_ has a hardness of approximately 919 HV and is suitable for use as a solidifying agent for alloys [102]. With a 2.0 wt. % addition, the alloy hardness was shown to increase by 22%, reaching 1163 HV. However, with a further increase in the additional amount, the hardness of the alloy will once again decrease. Furthermore, the compressive strength and bending strength of the alloy showed a nonlinear increase as the Mo_2_TiAlC_2_ content increased. At a content of 3.0 wt. %, they reached 3388 MPa and 823 MPa, representing an 18% and a 54.4% rise, respectively. The enhancement in alloy strength is mainly due to grain refinement. As depicted in Figure 4a, Mo_2_TiAlC_2_, which is predominantly localized at the grain boundary, can refine the grain size of both the Mo_ss_ phase and the intermetallic phase concomitantly, but the grain refinement will have an upper limit with the increase in its content. Therefore, it is reasonable to assume that the nonlinear increase in compressive strength and bending strength should be limited to the three test samples of Lin [99].

**Figure 5:**
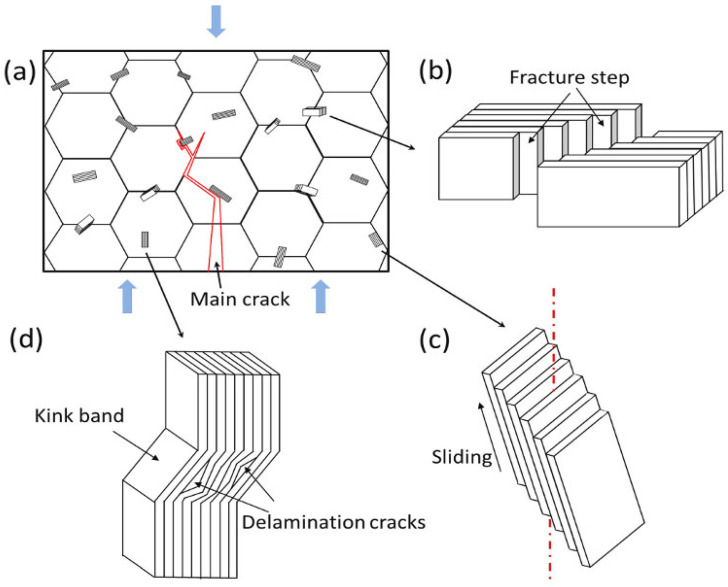
Schematic diagram of toughening mechanism of Mo_2_TiAlC_2_ relative to Mo-12Si-8.5B alloy: (**a**) Crack tip will deflect; (**b**) Steps formed on the fracture surface under the stress; (**c**) Interlayer sliding; (**d**) Kink-band formation [99].

After careful analysis, Lin concluded that there are three main reasons for the alloy toughening, and Figure 5 was drawn as a schematic illustration. Firstly, grain refinement reduced the length of cracks in alloys, which, according to Griffith’s flaw theory, increased the critical stress required for crack propagation. In addition, the crack tip would further consume its expansion energy when it encounters Mo_2_TiAlC_2_ to produce deflection (as shown in Figure 5a). Secondly, the distinctive layered structure of the Mo_2_TiAlC_2_ phase resulted in the formation of a stepped pattern during fracture, leading to a wider fracture area that required increased fracture energy (see Figure 5b). These continuous stepped structures were visible in the fracture morphology of the alloy shown in Figure 5b. Thirdly, when the stress angle deviated from the MAX phase base plane, adjacent base planes may have experienced an interlayer slip, thereby alleviating the stress on the surface of Mo_2_TiAlC_2_ particles and resulting in a slip step (see Figure 5c). Figure 5d displays the kink band resulting from a layered crack, although this was not observed in the experiment. Similar research has demonstrated that this formation effectively consumes energy during crack propagation, ultimately enhancing the fracture toughness of the material [103,104].

By summing up the above, it can be found that both doped ZrB_2_ and Mo_2_TiAlC_2_ particles have equivalent impacts on the mechanical properties. On the one hand, the greater the amount of doping in a certain range, as grain refinement becomes more favorable, compressive strength exhibits a monotonic increasing trend. However, there is an upper limit to grain refinement, so too high a doping amount is not useful for strength enhancement. Furthermore, based on the current research, when the doping amounts of ZrB_2_ and Mo_2_TiAlC_2_ reached 2.5 wt. % and 3.0 wt. %, respectively, the improvement in alloy strength reached its limit value. On the other hand, there is an optimal content for enhancing the toughness by doping both types of particles. This may be because when the grain size is excessively reduced, increasing the ratio of grain boundaries and phase boundary volumes can lead to a change in fracture mechanism and thus reduce the alloy toughness. This can be exemplified by the fracture mode switching of alloys with different amounts of La_2_O_3_ doping, but research on the fracture mode of ZrB_2_- and Mo_2_TiAlC_2_-modified alloys is lacking. The enhanced effects of doping the three particles mentioned above on the mechanical properties of the Mo-12Si-8.5B alloy are listed in Table 2, and doping Mo_2_TiAlC_2_ was found to have the best reinforcing effect on the strength and toughness of the alloy, but so far no researchers have investigated its oxidation resistance.

## 5. Effect of Si/B Ratio on Mo–Si–B Alloys

The properties of an alloy are influenced by the different phases present and their respective proportions. Research on the Mo–Si–B alloys originated from Akinc’s innovative addition of B to Mo_5_Si_3_, which reduced the viscosity of SiO_2_, formed more protective borosilicate (SiO_2_∙B_2_O_3_), and enhanced the overall coverage of oxidation scale on the substrate [105,106]. The idea of solving the problem of high-temperature strength but poor oxidation resistance of intermetallic compounds is given. Due to the superior ductility of Mo_ss_, with an acceptable balance between the high-temperature creep resistance and oxidation resistance and the room temperature fracture toughness of Mo_ss_-Mo_3_Si-T2 system alloys, it is more suitable for engineering and industrial applications. In this system, increasing the volume of intermetallic compounds can enhance the alloy strength and oxidation resistance at high temperatures, but will lead to a reduction in alloy toughness [35]. However, the low density and oxidation resistance largely depend on the high silicon content of its alloys, which inevitably hurts its fracture toughness [32]. Therefore, achieving a balance in the amounts and distribution of the three phases is a significant scientific challenge in producing Mo–Si–B alloys with optimal overall properties.

**Figure 6:**
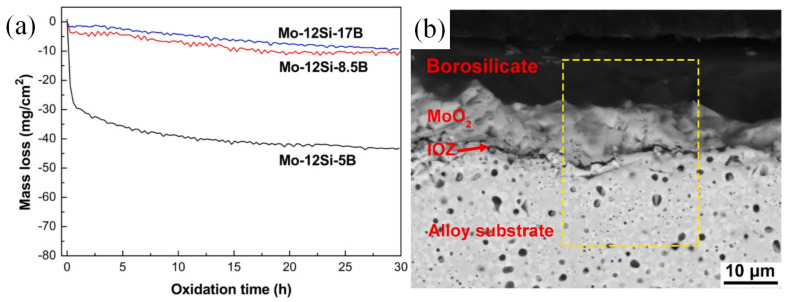
(**a**) Mass loss curves of three samples oxidized in 1000 °C air for 30 h; (**b**) BSE image of a cross-section of the Mo-12Si-17B sample. The dotted yellow box shows the EDS mapping page [107].

Li et al. changed the relative contents of the three phases by increasing the content of B. With the increase in the content of B, the diffraction peak of Mo_5_SiB_2_ was enhanced, while the other two phases were weakened [107]. Figure 6a reveals that, following an oxidation test at 1000 °C for 30 h on three different alloys with varying B contents, Mo-12Si-17B and Mo-12Si-8.5B displayed reduced mass loss during the transient oxidation stage due to the creation of a sleek and compact borosilicate layer. As depicted in Figure 6b, the oxide scale above the substrate consisted of the outermost layer of borosilicate, the underlying layer of MoO_2_, and the inner oxide zone (IOZ). MoO_2_ is produced by the reaction of Formula (6) after oxygen slowly diffuses inward through the borosilicate layer, while the IOZ layer consists of some silicon-rich phases. The formation of the inner oxide layer occurs due to the continuous diffusion of oxygen through the borosilicate layer. If the borosilicate fails to effectively limit the internal oxygen diffusion, it may lead to the thickening of the inner oxide zone. Therefore, Li examined the oxidation resistance of the borosilicate scale according to the thickness of the inner MoO_2_ layer and IOZ layer. Table 1 displays the fracture toughness (K_q_) and oxide thickness of three specimens varying in B content. As B content increased, the thickness of the outer borosilicate layer, the inner MoO_2_ layer, and the IOZ layer all decreased significantly. This indicates that the borosilicate layer in the outer layer of the Mo-12Si-17B sample, as a high-quality diffusion barrier, prevented oxygen molecules from diffusing inward, and had the best protection performance and stability. Furthermore, the fracture toughness showed a slight decrease with higher B content, but the increase in T2 phase content did not lead to significant deterioration.

**Figure 7:**
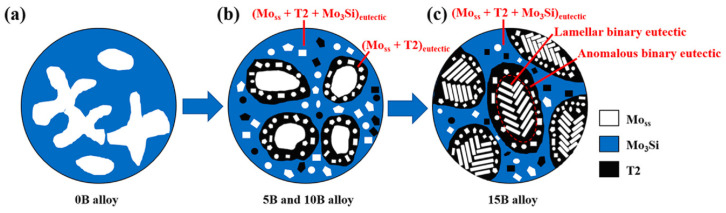
Microstructure evolution diagram of Mo-10Si-xB alloy: (**a**) x = 0; (**b**) x = 5, 10; (**c**) x = 15 [108].

**Figure 8:**
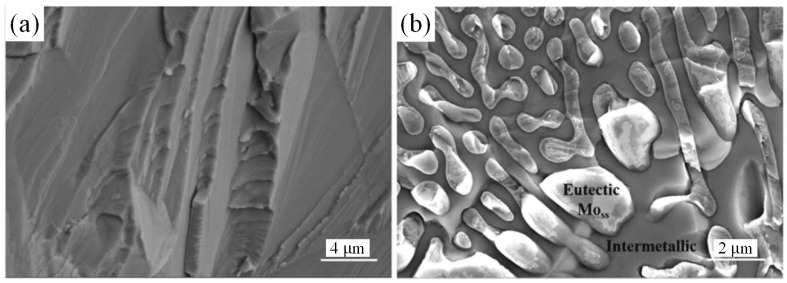
The fracture morphology of the sample: (**a**) Mo-10Si-0B; (**b**) Mo-10Si-15B [108].

Jin et al. studied the mechanical properties and high-temperature oxidation resistance of a Mo-10-xB (x = 0, 5, 10, 15) alloy [108], and summarized the microstructure evolution process of the alloy in combination with the sketch diagram shown in Figure 7. When x = 0, the alloy was in the Mo_ss_ + Mo_3_Si two-phase region, and the primary Mo_ss_ dendrites were surrounded by peritectic Mo_3_Si. The solidification sequence is given by Equation (12). When x = 5, 10, 15, the alloy was located in the three-phase Mo_ss_ + Mo_3_Si + T2 region. For x = 5, 10, the solidification path of the alloy can be expressed as Equation (13). First, Mo_ss_ was mainly precipitated, and the remaining melt solidified along the Mo_ss_-T2 binary eutectic valley. For x = 15, the alloy exhibited a high concentration of B, and the Mo_ss_-T2 binary eutectic was precipitated directly at the initial stage. The alloy was mainly composed of Mo_ss_-T2 binary eutectic and Mo_ss_-T2-Mo_3_Si ternary eutectic, whose solidification sequence can be expressed as Equation (14). Previously, to investigate the phase transition and solidification reactions of Mo–Si–B alloys, Kazemi utilized thermodynamic data from Factsage^TM^ to conduct phase-field simulations with Micress^TM,^ and confirmed the existence of binary and ternary eutectic reactions [7]. The alloy hardness increased with B content, reaching 1078.32 ± 59.9 HV at x = 15. However, the alloy experienced a significant reduction in fracture toughness. Upon Jin’s analysis of the fracture mode of the alloy, it was discovered that when the B content is low (x = 0 or 5), a cleavage surface appears on the fracture, displaying a typical transgranular fracture morphology, as depicted in Figure 8. However, with a larger B content (x = 15), there were bulging particles in the cross-section, indicating a shift to a mixed fracture mode, encompassing both intergranular and transgranular fractures. Due to its larger grain size, Mo_ss_ exhibited susceptibility to transgranular fracture with plastic deformation, while intergranular fracture with interface stripping occurs more frequently at the intermetallic phase and Mo_ss_ interface. The above-mentioned fracture pattern transformation is primarily triggered by an increase in T2 phase content. Li and Jin reached the same conclusion about the antioxidant activity of samples with different B contents [107].

L→L_1_ + (Mo_ss_)_Ⅰ_→(Mo_ss_)_Ⅰ_ + (Mo_3_Si)_peritectic_ (12)

L→L_1_ + (Mo_ss_)_Ⅰ_→L_2_ + (Mo_ss_)_Ⅰ_ + (Mo_ss_ + T2)_eutectic_→(Mo_ss_)_Ⅰ_ + (Mo_ss_ + T2)_eutectic_→(Mo_ss_ + T2 + Mo_3_Si)_eutectic_ (13)

L→L_1_ + (Mo_ss_ + T2)_eutectic_→(Mo_ss_ + T2)_eutectic_ + (Mo_ss_ + T2 + Mo_3_Si)_eutectic_ (14)

It is possible to adjust the phase ratio in the alloy by changing the content of B. Increasing the B content may increase the content of the ideal T2 phase content in the alloy, and the oxidation resistance of the alloy is significantly increased, but the alloy toughness will be slightly decreased, and the decrease in ductile phase Mo_ss_ content accounts for the toughness reduction. Therefore, in future research, it is necessary to focus on how to avoid the reduction in alloy toughness. Byun prepared a composite core–shell powder with a nanometer-scale molybdenum shell and an intermetallic compound core, and prepared the sample with a uniform distribution of the intermetallic compound in the continuous Mo_ss_ substrate by pressureless sintering [109]. Notably, even in pressureless sintering, a 3.3% porosity alloy sample was obtained; thus, further combination with the preparation method conducive to enhancing alloy densification holds the hope of achieving an alloy with a uniform distribution of microstructure and high density, which will greatly enhance the mechanical properties of the alloy. Fortunately, Guo reported a novel technique-oscillatory pressure sintering-for preparing Mo–Si–B alloys [2], whose basic principle is to superimpose a large constant pressure with a tunable frequency and amplitude of oscillatory pressure, which can greatly enhance the degree of alloy densification. The relative density of the alloy is as high as 97.78% at the 9 Hz oscillation frequency, which is 6% higher than that of the hot-pressed sintered alloy, and the fracture toughness is also significantly improved.

## 6. Effect of Bimodal Mo_ss_ Structure on Mo–Si–B Alloys

Grain size plays a significant role in determining the mechanical properties and oxidation resistance of Mo–Si–B alloys. On the one hand, finer grains result in higher strength as a result of the Hall–Petch effect, and the fine-grained structure allows for the rapid coating of the borosilicate scale, which improves the oxidation resistance of the substrate [3,30,86]. On the other hand, the storage capacity of dislocations in fine grains is limited, resulting in the low ductility and fracture toughness of the alloy. In addition, for high-temperature structural materials, the coarser the microstructure, the stronger the creep resistance [110]. An excessively coarse or fine microstructure can lead to deficiencies in one or more aspects of an alloy’s performance. Therefore, the focus is on achieving a balancing act by adjusting the microstructure size to enhance alloys' comprehensive properties. To enhance the overall characteristics of the alloy, Wang et al. synthesized Cu with a bimodal grain size, comprising micro-sized grains incorporated into nano-sized grains, resulting in increased strength and ductility [111]. Subsequent research revealed that larger, coarse grains exhibit enhanced ductility due to strain hardening, while finer, nano-sized grains are associated with higher strength. The combination of the two can yield an optimal balance of strength and toughness. Significantly, several experiments have demonstrated that the presence of a bimodal structure does not affect the phase composition and ratio of the alloy [112,113,114].

**Figure 9:**
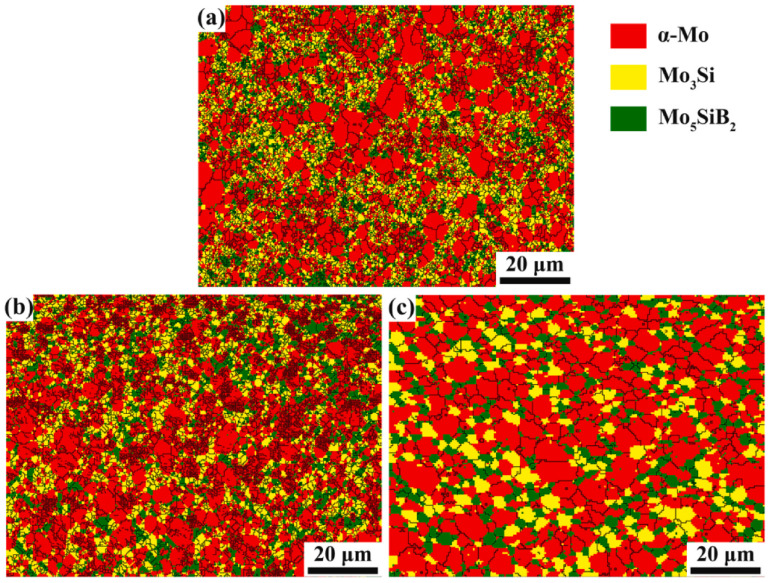
EBSD microscopic image of bimodal alloy: (**a**) Unannealed treatment [36]; (**b**) annealing treatment at 1700 °C; (**c**) annealing treatment at 1800 °C [117].

At present, the main manufacturing method is to adjust the ratio of fine powder to coarse powder and to use powder metallurgy technology to produce bimodal structural alloys [36]. For example, Li et al. prepared a Mo-12Si-8.5 alloy with a bimodal Mo_ss_ structure doped with La_2_O_3_ particles (0.57 wt. %) and increased its fracture toughness up to 12.5 MPa∙m^1/2^, and its yield strength and compressive strength were found to be as high as 2460 MPa and 2561 MPa [36]. The bimodal alloys are strengthened by passivation and cracks trapping by the coarse-grained Mo_ss_ microstructure. However, the uneven dispersal of a fine and coarse-grained microstructure has a detrimental impact on the continuity of Mo_ss_, thereby decreasing the probability of crack capture, which is the key problem limiting the enhancement of alloys fracture toughness. Given that annealing is an effective method to increase grain size and increase structural homogeneity [115,116], Li conducted annealing on the Mo–Si–B bimodal alloys at temperatures of 1700 °C and 1800 °C, based on previous research, to further optimize their mechanical properties [117]. As shown in Figure 9, the distribution of the Mo_ss_ phased in the unannealed alloy was unevenly piled up. However, after annealing at 1700 °C, the degree of grain coarsening in the fine-grain region varies, and accumulation in the coarse-grain region was weakened. Furthermore, when the annealing temperature was raised to 1800 °C, dispersed micron-sized Mo_3_Si/T2 particles were observed in a continuous bimodal Mo_ss_ phase. On the one hand, following annealing, grains in both fine and coarse regions experienced coarsening, leading to improved microstructure uniformity and an increased volume fraction of Mo_ss_ distribution, which enhanced the toughening effect of crack trapping and helped reduce the driving force of crack growth. On the other hand, the pinning mechanism of La_2_O_3_ particles nested in Mo_ss_ causes dislocation to accumulate on one side of the particles, and causes an increase in internal stress [118], resulting in micro cracks around the main crack, which also reduces the driving force of crack growth. Two factors contribute to the enhancement of fracture toughness in the alloy after annealing at 1800 °C, increasing from 9.2 MPa∙m^1/2^ to 13.41 MPa∙m^1/2^. However, the general coarsening of the alloy grains leads to a reduction of 12.2% in compressive strength when compared to the fine-grained alloy [36]. To this end, Li further investigated the compression characteristics of the bimodal alloy within the temperature range of 1000–1400 °C. At 1000 °C, both the compressive and yield strength of the bimodal alloy were lower than that of the fine alloy, as the micrometer Mo_ss_ coarse grains weaken grain boundary strengthening. However, at temperatures of 1200 °C and 1400 °C, the bimodal alloys exhibited higher compressive and yield strengths because of the little grain boundary sliding deformation in the coarse-grained region. In particular, at 1400 °C, a significant strength improvement can be attributed to work hardening resulting from extensive plastic deformation in the Mo_ss_ coarse-grained region. Moreover, the presence of La_2_O_3_ particles within and between grains enhanced the strength of the alloy through the suppression of dislocation movement and the stabilization of grain boundaries.

Wang utilized the same technique to fabricate a Mo-12Si-8.5B bimodal microstructure alloy that was doped with 1.0 wt. % ZrB_2_, and the toughening effect was consistent with the above [119]. She summarized the reinforcement mechanism as Mo_ss_ phase and intermetallic phase intrinsic reinforcement, grain boundary reinforcement, and Orowan reinforcement. Subsequently, she further investigated the oxidation resistance of the alloy at 1100 °C, and the volume fraction of coarse grains above 1 μm increased with the increase in the unmechanically alloyed powder added [120]. The MSBZ-50 alloy comprises unmechanically alloyed powder, which contributes to 50% of its mass fraction, and the average grain size of both coarse and ultra-fine Mo_ss_ increases to 2.25 μm and 0.55 μm, respectively. The area fraction of coarse Mo_ss_ constitutes 58.5% of the total area fraction of Mo_ss_. The weight loss of the MSBZ-50 alloy with a higher proportion of coarse grains was more significant, and oxidation persisted even after entering the steady-state oxidation phase. Nevertheless, it was satisfactory that the weight reduction in MSBZ-20 alloy with an unmechanical alloying powder ratio of 20% decreased by 62% during the transient period compared to the ultra-fine alloy UFG-MSBZ [119]. The Mo_ss_ coarse and fine grains in MSBZ-20 exhibited average sizes of 2.15 μm and 0.45 μm, respectively, with a volume fraction ratio of approximately 1:1.7. Compared to the SEM cross-section images of the three alloys that were oxidized at 1100 °C for 30 h, the oxide layer on MSBZ-20 was thinner, while the grain boundaries of the UFG-MSBZ alloy were more distinct due to its finer grain structure. During the initial oxidation stage, the grain boundaries act as channels for oxygen diffusion, so the transient oxidation rate of the UFG-MSBZ alloy was larger than that of MSBZ-20. However, the larger grain size of the borosilicate layer results in a longer transverse diffusion distance, resulting in MSBZ-20 having a slower steady-state oxidation stage. The mechanical properties of the fine grain alloy doped with the second phase particles and the bimodal alloy are compared in Table 2.

## 7. Mo–Si–B Alloy Made by Additive Manufacturing

The process for preparing Mo–Si–B alloys typically involves arc melting, powder metallurgy, and additive manufacturing [46]. Arc melting has the advantage of low input energy and high synthesis speed, but it often results in the formation of micro and macro cracks during the manufacturing process [121]. Alloys manufactured by powder metallurgy have problems such as high porosity and more macroscopic cracks [122,123]. Although the relative density of alloys is close to 96% through improvement, it is still difficult to obtain nonporous bulk materials [124]. More importantly, even with high static pressure, the phenomenon of powder agglomeration cannot be effectively disrupted. Compared to these two traditional manufacturing processes, additive manufacturing offers design freedom, reduces production steps, and enables the net formation of complex structural parts. Therefore, it has promising applications in the production of metal parts with complex structures and shapes [125,126,127,128,129]. At present, the use of additive manufacturing for the production of a Mo–Si–B alloy is still in the early stages of investigation.

Schmelzer demonstrated for the first time the feasibility of printing Mo–Si–B alloy powder materials by direct energy deposition (DED) [130]. Later on, Becker utilized DED to produce a Mo-9Si-8B alloy, and studied its oxidation resistance ranging from 800 to 1300 °C. Due to the coarse grain size of Mo_ss_ and the low T2 phase content in alloys processed by DED, the “pesting” oxidation phenomenon was more serious than that in alloys manufactured by powder metallurgy. Laser powder bed fusion (LPBF), also known as selective laser melting (SLM), is an additive manufacturing technology that selectively melts the metal powder and produces parts based on a 3D CAD model using a laser system. This technology has been successfully applied to produce alloys such as Al [131], Ti, and Ni [132,133]. After verifying the feasibility of preparing Mo [134,135], Makineni et al. used LPBF to create Mo–Si–B alloys doped with La_2_O_3_ particles [136]. The supercooling environment of the LPBF process effectively prevented the formation of Mo_3_Si phases, resulting in the formation of the alloy with a phase composition of Mo_ss_ + Mo_5_SiB_2_ + Mo_5_Si_3_. Yoshimi et al. developed a ceramic reinforced system alloy with a phase composition of 65Mo-5Si-10B-10Ti-10C, which has excellent high-temperature creep properties, a room temperature fracture toughness up to 15 MPa∙m^1/2^, and density comparable to or even lower than nickel-based superalloys [137,138,139]. In addition, the MoS–BTiC alloy also exhibits the characteristics of high brittleness, high melting point, and difficult processing. In an attempt to utilize additive manufacturing technology for alloy preparation, Zhou et al. employed a combination of high-energy ball milling and screening to produce an alloy powder with controllable particle size and uniform flow [140]. They then used LPBF technology to produce the MoSiBTiC multiphase alloy for the first time, which boasted finer grains and higher uniformity than the as-cast alloy. However, the high thermal gradient generated during the rapid cooling phase of the LPBF manufacturing process can lead to severe thermal stress within the specimen, which can lead to a large number of microcracks in the alloy [141,142]. For this reason, the mechanical properties of LPBF alloys are poorer than those of as-cast alloys. Hot isostatic pressing (HIP) can solve this problem. However, it can alter microstructure homogeneity and induce phase changes when enhancing the quality of additive manufacturing products [143,144,145]. To this end, Zhou also studied the effects of HIP on the microstructure and fracture toughness of the alloy at room temperature [146]. In the rapid cooling phase, crack formation and expansion will occur first in the brittle phase aggregation region. The rearrangement and sliding of grain boundaries after HIP treatment will make the microstructure of the alloy more uniform and denser, and the surface microcracks will be bridged after TiC precipitation at high temperatures. This significantly reduces the number of cracks, and the length of the crack also decreases as HIP temperature increases. The strengthening of the ductile phase aids in accommodating strain and inhibiting cracks. Following the 1700 °C HIP treatment, the alloy fracture toughness increased to 9.0 MPa∙m^1/2^, yet the alloy hardness decreased. Takeda et al. investigated the tensile and compressive behaviors of Mo-5Si-10B-10Ti-10C alloy produced by LPBF. The authors observed that while HIP treatment improved the alloy elastic and strength properties, the mechanical properties were still suboptimal compared to those of the as-cast alloy, mainly because HIP failed to completely eliminate cracks and pores in the alloy. Furthermore, Takeda discovered that the alloy exhibited significantly different macroscopic mechanical reactions when subjected to tensile and compressive forces, as the propagation and closure of the microcrack varied according to the applied load and direction of the crack.

**Figure 10:**
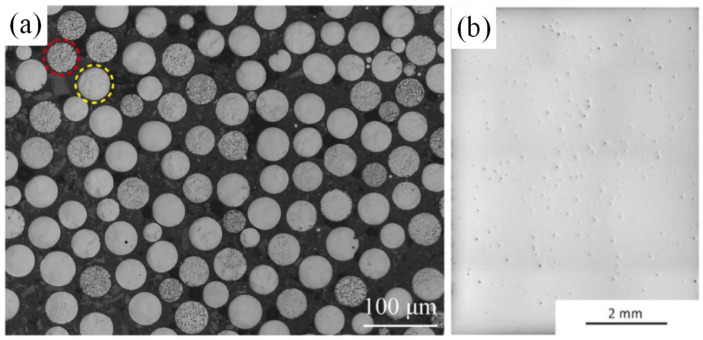
(**a**) Optical microscopic image of particle cross section of plasma spheroidized powder (Yellow-dot circles indicate featureless particles, and red-dot circles indicate precipitated particles) [149]; (**b**) sample prepared after powder heat treatment [150].

The metal powder utilized in the LPBF method requires a spherical shape, optimal fluidity, and uniform particle size [134,135]. However, due to the alloy complex phase composition and high melting point, obtaining such a powder presents a challenging task. At present, it is difficult to prepare spherical Mo-base alloys powder with controllable size using mature aerosol technology [147,148]. High-energy ball milling has been proven to refine particle size and produce consistent powder, but the process involves repeated deformation, fracturing, and cold welding of the powder, which can cause significant damage to its quality. Alloy powders commonly utilized in powder metallurgy and additive manufacturing inevitably contain oxygen that leads to harmful oxide impurities. Therefore, the production of high-quality, clean powders remains a crucial aspect of material manufacturing. The oxygen content in powder is greatly affected by the particle size and increases with the decrease in particle size, and the removal of ultrafine particles (<20 μm) is beneficial to reducing the oxygen content in the powder. To obtain an alloy powder that meets the manufacturing requirements, Higashi and Ozaki prepared spherical powder particles by plasma spheroidization with only a few pores in the particles, as shown in Figure 10a [149]. However, the mechanical properties of the alloy samples made of this powder were still not ideal. The high melting point of the Mo and the rapid cooling during LPBF processing will enable the sample to withstand high thermal stress during the manufacturing process, and the mixture of impurities such as nitrogen and oxygen will significantly affect the toughness and brittleness transition temperature of the finished product, which can easily lead to cracks. Therefore, another key problem in the production of Mo–Si–B alloys via LPBF is reducing crack density within the sample. HIP treatment has a limited impact on crack mending. However, preconditioning the fabricated plate can significantly decrease the cooling rate. Fichtner et al. combined improved powder quality with the preheating of the constructed plate to reduce the formation of microcracks during the manufacturing process [150]. To ensure powder quality, a ring shear test was used to test powder fluidity before processing. To minimize impurities such as oxygen and nitrogen during powder preparation, the powder was subjected to heat treatment in a flowing argon environment. The resulting sample displayed no cracks, but still contained a small number of pores, as demonstrated in Figure 10b. The LPBF process involves many parameters, and optimizing these parameters is an important topic. Ma et al. studied the influence of LPBF process parameters on the relative density of the alloy by orthogonal testing, and found that the layer thickness had the greatest influence, followed by laser power and hatch spacing, and scanning speed had the least influence [151]. Then, Mo–Si–B alloys with a relative density of 99.2% were prepared after the process parameters were optimized, corresponding to the above parameters of 20 μm, 500 W, 30 μm, and 700 mm/s respectively. During the LPBF manufacturing process, grain growth along the construction direction tended to form columnar crystals, and the average grain size of the longitudinal section (38.57 μm) was larger than that of the transverse section (11.87 μm). In addition, the relatively regular cavities in the bulk alloy were mainly due to the pores in the powder, and the powder was not fully fused; MoO_3_ will form small and regular pores after evaporation.

Based on the analysis above, it is evident that the high porosity of the alloy samples produced by L-PBF is a key issue that impedes the enhancement of their performance. To resolve this issue, the first step is to enhance the quality of the powder to guarantee its purity and the uniformity of its particle size. The second step is to consider the manufacturing process, which can be optimized from the construction parameters and the use of substrate preheating means, and the current layout of the matrix and powder for Mo–Si–B alloys is still in the design optimization stage, thus requiring further process advancements in the future. Finally, the sample may be HIP-treated. However, the effectiveness of HIP in reducing alloy porosity varies greatly, primarily due to the low solubility of Ar in the metal [152,153,154]. Shao et al. investigated the Ar formation energy in pure titanium using density functional theory, and suggested that pores with initial sizes below 1 μm are more easily eliminated by HIP, requiring lower temperatures, pressures, and treatment times [155]. Therefore, for the future use of HIP post-processing to improve the quality of finished products, it is necessary to establish standards in combination with various variable factors, and optimize the build parameters. In this way we can enhance the quality of powders employed in additive manufacturing and optimize the substrate heating procedures.

## 8. Outlook

Based on the above analysis, it has been identified that the overall characteristics of the Mo–Si–B alloys are hampered by the distinct attributes of each phase, which remains a pressing issue that requires resolution. Currently, modifications involving the doping of single metal elements and second-phase particles have proven to be effective in specific areas, but they cannot comprehensively enhance the properties of alloys. Although MAX phase doping can significantly improve the mechanical properties of the alloy, its effect on the oxidation resistance of the alloy is still unknown. Furthermore, while a bimodal structure design may positively impact the mechanical properties, it may not be beneficial and may even damage oxidation resistance. Xie’s oscillatory sintering technology has yielded high-density zirconia ceramics of up to 99.7% [156,157]. Although this technology has shown promising results in improving the density of Mo–Si–B alloys [2], it has not received enough attention. Additive manufacturing technology is an essential direction for material preparation; however, due to the high melting point and hardness of Mo-based alloys, an efficacious solution is yet to be found for the problem of the production of numerous cracks and holes in the Mo–Si–B alloys. Considering these discussions and challenges, this paper proposes research directions worth considering to further enhance the performance and practicality of Mo–Si–B alloys:

(1) Alloy composition formula design. The properties of Mo–Si–B alloys with different phase compositions have been studied, and the undesirable phase Mo_3_Si can be avoided by adjusting the composition ratio. This is combined with polymetallic elements and second-phase particles’ modification to achieve the balance of mechanical properties and antioxidant properties. By incorporating ZrB_2_, the viscosity of borosilicate can be decreased rapidly to efficiently coat the sample surface and establish a durable protective layer through the passivation of Zr in borosilicate. As a result, further research could explore the impact of B-rich particles on enhancing the oxidation resistance of Mo–Si–B alloys. Furthermore, the potential advantages of the MAX phase and Ti doping to enhance the mechanical properties and reduce the density of Mo–Si–B alloys are a direction that needs further investigation;
(2) The oscillatory sintering process is a potent means of increasing the density of the alloy, resolving the issue of powder agglomeration, and rendering uniform the microstructure phase distribution of the alloy. Consequently, the amalgamation of bimodal structure design, doping modification, and the oscillatory sintering process holds great promise for imparting to the alloy excellent comprehensive properties;
(3) Currently, the ceramic cores used in the preparation of turbine blades face issues such as inhomogeneous heating and low brittleness, which pose challenges in creating gas film holes that are less than 0.5 mm in size and advanced cooling structures. While using Mo-based refractory alloys in place of nickel-based alloys for preparing turbine blades presents many problems, the requirements for the mechanical properties of the core are not strict. By designing suitable coatings, the oxidation resistance of a Mo-based refractory metal core can be addressed, enabling its high melting point to be fully utilized in the preparation of efficient air-cooled blade cores, so as to further improve the cooling efficiency of the blade, which is of great significance in relation to improving the gas temperature in the front of the turbine.

## Figures and Tables

**Table 1:** Fracture toughness and oxide thickness of samples with different B contents [107].

	Mo-12Si-5B	Mo-12Si-8.5B	Mo-12Si-17B
Fracture toughness (K_q_)/MPa m^1/2^	9.8	9.3	8.7
Outer borosilicate layer/μm	43.2	14.8	11.3
Inner MoO_2_ layer/μm	200.0	37.5	8.4
Inner Oxidation zone (IOZ)/μm	13.5	8.4	2.9

**Table 2:** Mechanical properties of doped second-phase particles and bimodal and fine-grained alloys.

Mo-12Si-8.5B	Grain Structure	Compressive Strength/MPa	Fracture Toughness/MPa∙m^1/2^
+xLa_2_O_3_	Fine (x = 0.9 wt. %) [91]	2807	9.3
	Fine (x = 0.57 wt. %) [36]	3058	9.2
	Bimodal (x = 0.57 wt. %) [36]	2561	12.5
	Bimodal (x = 0.57 wt. %) [117]	2681	13.41
+xZrB_2_	Fine (x = 1.0 wt. %) [22]	3130	11.5
	Bimodal (x = 0.57 wt. %) [119]	2998	13.1
+xMo_2_TiAlC_2_	Fine (x = 2.0 wt. %) [99]	3356	14.07

## Data Availability

No new data were created or analyzed in this study. Data sharing is not applicable to this article.
